# Effect of mindfulness group therapy on maternal psychological distress and perinatal outcomes in twin pregnancy: a randomized controlled trial

**DOI:** 10.3389/fpsyt.2025.1572633

**Published:** 2025-05-20

**Authors:** Ying Zhou, Zai-Mei Tang, Lin Mei, Xiao-Chang Yang, Hong-Ling Zheng, Bi-Zhen Liao, Xin-Yang Yu

**Affiliations:** ^1^ Department of Obstetrics and Gynecology, Chongqing Key Laboratory of Maternal and Fetal Medicine/Joint International Research Laboratory of Reproduction & Development, Ministry of Education/The Innovation and Talent Recruitment Base of Maternal-Fetal Medicine, The First Affiliated Hospital of Chongqing Medical University, Chongqing, China; ^2^ School of Marxism, Chongqing University, Chongqing, China; ^3^ Department of Gastrointestinal Surgery, The First Affiliated Hospital of Chongqing Medical University, Chongqing, China

**Keywords:** twin pregnancies, depression, anxiety, stress, mindfulness, perinatal outcomes

## Abstract

**Purpose:**

Psychosocial stress, depression, and anxiety are prevalent during pregnancy and can be associated with adverse birth outcomes. This study aimed to evaluate the effectiveness of an online Mindfulness Group Therapy (MGT) program in improving perinatal maternal psychological distress and perinatal outcomes among women with twin pregnancies.

**Methods:**

In this randomized controlled trial, 120 women carrying twins were assigned to MGT groups (6-week, 120 minutes of mindfulness intervention weekly) or control groups (usual perinatal care and health education). The primary outcomes were maternal psychological distress, including depression, anxiety, stress, and mindfulness measured by the Edinburgh Postnatal Depression Scale, State Anxiety Inventory, Perceived Stress Scale, and Five Facet Mindfulness Questionnaire at three points: baseline, post-intervention and time one month postpartum. Secondary outcomes included maternal pregnancy outcomes and neonatal outcomes.

**Results:**

A total of 109 women with twin pregnancies completed the intervention. The intervention significantly and effectively prevented the worsening of postpartum depression symptoms in women with twin pregnancies, improved mindfulness, and reduced perceived stress (P < 0.05). Significant differences were observed at both post-intervention, and 1-month post-delivery (P < 0.05). However, no significant differences were found in anxiety scores (P > 0.05). Intention-to-treat analysis further revealed that the intervention had a significant effect on reducing postpartum depressive symptoms (P < 0.05), even when considering participants who did not complete the entire study process. In terms of pregnancy outcomes, a significant difference was found between the intervention and control groups in the incidence of low birth weight (P<0.05).

**Conclusion:**

MGT proves potentially effective in reducing perinatal stress, preventing prenatal depression, and decreasing the incidence of low-birth-weight infants in twin pregnancies. These findings support the integration of group mindfulness interventions into prenatal mental health care to mitigate prenatal depression among women expecting twins.

**Clinical Trial Registration:**

https://www.chictr.org.cn/showproj.html?proj=131787, identifier ChiCTR2100050091.

## Highlights

- This inaugural study developed and evaluated mindfulness group therapy tailored specifically for pregnant women with twins.
- Mindfulness group therapy supports pregnant women with twins in preventing the exacerbation of depressive symptoms.
- The benefits of group mindfulness therapy may persist into the postpartum period.
- Mindfulness group therapy is associated with a lower incidence of low-birth-weight infants among twin pregnancies.

## 1 Introduction

Assisted reproductive technology is now widely used, significantly increasing the incidence of twin pregnancies (1). Twin pregnancies are considered high-risk, with substantially higher rates of neonatal morbidity and mortality (2). Significant physiological changes, mobility limitations and bed rest (3), with doubled economic pressures and caregiving demands (4), elevate psychological stress in women with twin pregnancies. This stress may heighten the risk of psychological disorders such as anxiety or depression (5). Women with twin pregnancies often endure additional stressors compared to those with singleton pregnancies, leading to increased stress levels and a greater risk of disorders like anxiety and depression (6). Previous studies have indicated that about 34.8% of women carrying twins experience anxiety symptoms and 37.1% suffer from depressive symptoms (7)—rates significantly higher than those observed in singleton pregnancies, which are 24.6% for anxiety (8) and 28.4% for depression (9).

Negative prenatal emotions are associated with adverse maternal and perinatal outcomes. Elevated stress levels in twin pregnancies may increase the risks of premature membrane rupture and preterm birth (10, 11). Higher depressive symptoms in twin pregnancies have been linked with a lower Apgar scores in newborn boys (12). A systematic review has shown that perinatal anxiety and depression can adversely affect the neurodevelopment of children and adolescents (13). Twin pregnancies may exacerbate poor psychological outcomes (14). There is evidence suggesting that postpartum depression may continue from prenatal depression (15), and women with high prenatal anxiety levels face a heightened risk of increased anxiety after delivery (16). Furthermore, untreated perinatal mental disorders impose significant economic burdens (17). Thus, poor prenatal mental health represents a crucial patient safety concern, a public health issue, and a preventable factor in maternal and infant mortality, deserving the attention of healthcare providers and policymakers (6). However, no studies to date have explored psychological interventions to improve adverse perinatal outcomes in twin pregnancies. This paper aims to fill this gap.

Since pharmacological treatments may provide safety risks during pregnancy (18), there is an urgent demand for non-pharmacological, evidence-based psychological interventions for women pregnant with twins. Mindfulness-based therapies (MBIs) positively impact maternal mental health by fostering non-judgmental awareness of the present moment (19). Mindfulness is the awareness of the present moment without judgment (20) and includes five dimensions: observing, describing, acting with awareness, non-judging of inner experiences, and non-reacting to inner experiences (21). The anterior cingulate cortex, amygdala, and insula are brain regions that control attention, memory, and emotions. Mindfulness practice can alter the activity levels of these brain regions, thereby enhancing an individual’s emotional regulation abilities (22). Systematic reviews and meta-analyses show that MBIs can reduce depression and anxiety symptoms in women with singleton pregnancies (23). MBIs, especially group programs, are structured, low-cost, and easy-to-implement methods that can be integrated into daily life (24). MBIs are less cognitively demanding, easier to self-practice at home, and adaptable to varying physical conditions for women with twin pregnancies. Thus, we adopted a group-based mindfulness intervention to alleviate stress, anxiety, and depressive symptoms in women with twin pregnancies.

While most studies on psychological interventions during pregnancy have focused on singleton pregnancies (25), no studies have specifically examined these interventions for maternal psychological distress in twin pregnancies. Therefore, this study aims to investigate the potential effectiveness of similar interventions in this unique subgroup of pregnancies. This study assesses the impact of MGT on mindfulness and stress levels, as well as anxiety and depression symptoms, and evaluates its effects on pregnancy outcomes in women expecting twins. Providing tailored healthcare to enhance mental health and improve perinatal outcomes for women pregnant with twins holds substantial social importance.

## 2 Materials and methods

### 2.1 Trial design and participants eligibility

This randomized, single-blind, single-center study comprised two parallel arms. Pregnant women with twins (20–28 weeks) were recruited at the First Affiliated Hospital of Chongqing Medical University, China, from September 2021 to December 2022. Adopt a multi-channel, phased recruitment strategy to ensure that a sufficient number of qualified participants are recruited. The recruitment process includes screening from hospital databases and also promoting through social media platforms. The eligibility of potential participants is assessed through questionnaires and retrospective reviews of electronic medical records, determined based on inclusion and exclusion criteria.

Inclusion criteria included being at least 18 years old, a confirmed intrauterine twin pregnancy via ultrasound, and fluency in reading Chinese. Exclusion criteria included suicidal ideation (EPDS item 10 score ≥ 1), severe organic or congenital diseases, serious mental disorders such as bipolar disorder or schizophrenia, plans for fetal reduction, stillbirth, fetal defects, and previous or current participation in mindfulness training. This study protocol was approved by the Ethical Committee of the First Affiliated Hospital of Chongqing Medical University (2020–199), and the trial was registered in the Chinese Clinical Trial Registry (ChiCTR2100050091) in August 2021.

**Figure 1:**
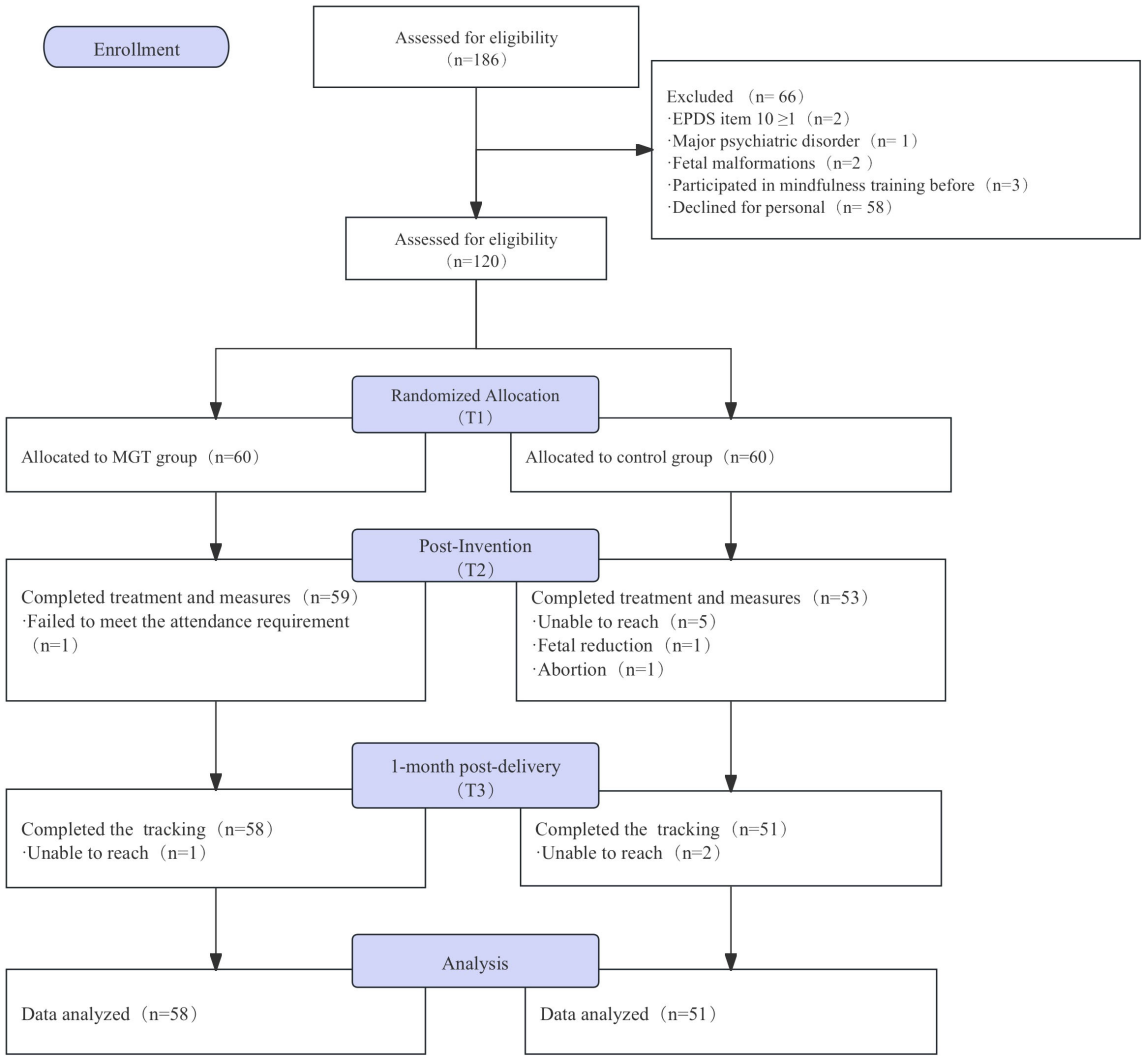
The CONSORT flow diagram for the study.

We adhered to the nonpharmacological treatment outlined in the Consolidated Standards of Reporting Trials (CONSORT) statement. A screening process assessed 186 Chinese women who were diagnosed with twin pregnancies; 66 were excluded for various reasons, including suicidal ideation, psychiatric diagnoses, fetal malformations, previous mindfulness training, and refusal to participate. Ultimately, 120 pregnant women were included in the study (**Figure 1**).

### 2.2 Sample size

The PASS 15 software was used to calculate the sample size based on the mean difference between the two groups. Depression, as measured by the Edinburgh Postpartum Depression Scale (EPDS), was the primary outcome in this study. Preliminary analysis from the first 24 participants indicated an average EPDS score of 8.00 in the intervention group and 8.45 in the control group. With α of 0.05 and a desired error margin of less than 0.7, a sample size of 51 participants per group was necessary. Considering a potential 10% dropout rate, the sample size was adjusted to approximately 57 participants pre group. Due to recruitment challenges and the structure of group therapy, a minimum of 12 participants per group was required, resulting in a total sample size of at least 120 participants.

### 2.3 Procedures

Block randomization was used in this study. Despite aiming to recruit 120 participants, recruitment challenges limited enrollment to 24 participants at a time. Participants were randomly assigned to the intervention group (n=12) or the control group (n=12) using SPSS 26 software. Group assignments were concealed in opaque envelopes, and participants selected envelopes sequentially by admission order. Participants were then randomly assigned to either the intervention group, which received a 6-week program of mindfulness intervention combined with group counseling, or control group, which participated in a 6-week WeChat health education program. Participants in both the intervention and control groups received standard routine medical care throughout the trial. The personnel who enrolled and those who assigned participants to interventions were unable to access the random allocation sequence. The outcome collectors remained blinded to the treatment allocation.

### 2.4 The intervention: mindfulness group therapy

Mindfulness Group Therapy (MGT) is based on the theoretical foundations of Mindfulness-Based Stress Reduction (MBSR) and Mindfulness-Based Cognitive Therapy (MBCT), aiming to alleviate pregnancy-related stress, anxiety, and depression in women pregnant with twins by cultivating mindfulness awareness. MBSR is the foundation of mindfulness interventions during pregnancy (26). MBCT was developed by incorporating cognitive psychology principles into MBSR. Previous studies have found that MBSR can alleviate depressive symptoms by cultivating mindfulness awareness, and it can also reduce anxiety and stress (27). The course content is based on the MBSR framework developed by Jon Kabat-Zinn and Nancy Bardacke’s *Mindful Birthing and Rearing* (28). Since pregnancy-related stress can indirectly increase symptoms of anxiety and depression by reducing social support (5), the intervention is delivered in a group format, providing participants with a structured platform for experience sharing, emotional support, and psychological well-being improvement. The intervention is delivered online to ensure convenience and accessibility for pregnant women, especially those with mobility restrictions due to twin pregnancies. We have specifically designed mindfulness interventions for women carrying twins, incorporating cognitive education. Women carrying twins typically engage in less activity than those carrying a single fetus, and by the late stages of pregnancy, they may need to remain in bed for extended periods (29). Our interventions combine mindfulness practices with gentle activities, such as seated or standing yoga stretches, to alleviate their physical and psychological stress.

The MGT program consists of six scheduled weekly sessions, each lasting 120 minutes (30). The course adopts a structured design to ensure the standardization of interventions and makes appropriate adjustments based on participant feedback. Each week, the program focusing on specific themes such as emotional awareness, stress management, interpersonal relationships, and continuous practice. Each session includes four parts: group activities, the main content of the lecture, formal mindfulness practices, and informal mindfulness practices. Group activities aim to build connection and encourage experience sharing. The core material of the presentation includes an introduction to relevant subjects. Formal mindfulness practices include mindfulness meditation, mindfulness breathing, awareness stretching, and body scanning; informal mindfulness practices include mindful eating, mindful walking, daily life stops, and mindful conversation. In the mindfulness breathing module, the therapist initially explains the principles and benefits of mindfulness breathing to the participants, subsequently demonstrates the correct execution of the practice, and finally guides the participants in engaging in the actual practice. The therapist conducts a session for participants to express their emotions and experiences during the practice while also addressing and guiding them on their questions. Taking mindfulness breathing as an example, the therapist initially explains the principles and benefits of mindfulness breathing to the participants, subsequently demonstrates the correct execution of the practice, and finally guides the participants in engaging in the actual practice. The therapist conducts a session for participants to express their emotions and experiences during the practice while also addressing and guiding them on their questions. Detailed content of the group mindfulness intervention course can be found in Appendix 1. After each session, simple homework is assigned to encourage participants to practice mindfulness for at least 20 minutes daily at home, with mindfulness guidance recordings provided as support.

The intervention is implemented by professionally qualified psychological counselor, who have received specialized training in mindfulness teaching. Researchers assist in the preparation and execution of the course, while psychiatrists provide full supervision and guidance. The course is conducted online, with researchers recording participants’ attendance. To increase participant adherence, the research team offers cash incentives and free fetal heart monitoring. Completion of the intervention was defined as participation in training for at least 5 weeks.

### 2.5 Data collection

#### 2.5.1 Basic information form

Collected data included the participant’s age, pre-pregnancy BMI, education level, residence, previous health status, depression history, parity, planned pregnancy, mode of conception, and chorionic type of twin pregnancy.

#### 2.5.2 Edinburgh postnatal depression scale

The Edinburgh Postpartum Depression Scale (EPDS) developed by Cox in 1987 (31), consists of 10 items scored from 0 to 3 on a Likert scale, for a total possible score of 0-30. A higher score indicates greater depression experienced by pregnant women in the past month. It is the most commonly used tool worldwide for assessing depression during pregnancy and postpartum (Cronbach’s α = 0.80) (32).

#### 2.5.3 State anxiety inventory

The State Anxiety Inventory (STAI-S), a subscale of the State-Trait Anxiety Inventory developed by Charles Spielberger et al. (33), assesses situation-specific anxiety. Comprising 20 items rated from 1 to 4, it yields a total score ranging from 20 to 80, with higher scores indicating more severe anxiety levels. The STAI-S is noted for its adequate internal consistency (Cronbach’s α = 0.95) (34).

#### 2.5.4 Perceived stress scale

The Perceived Stress Scale (PSS) was created by Dr. Cohen in 1983 (35), includes 14 items across dimensions: out-of-control and tension. Items are scored on a 5-point scale (0–4), including positively and negatively scored items, with a total score ranging from 0 to 56. Higher scores reflect greater psychological stress over the past month. The scale is widely used and has demonstrated good reliability and validity (Cronbach’s α = 0.81) (36).

#### 2.5.5 Five facet mindfulness questionnaire

The Five Facet Mindfulness Questionnaire (FFMQ) was developed by Baer et al. in 2011 (37). The scale has 39 items, each rated on a 5-point Likert scale (1–5), encompassing both positive and negative items. A higher total score indicates grater mindfulness. The FFMQ is extensively utilized in mindfulness research and exhibits good reliability and validity (Cronbach’s α ranges from 0.79 to 0.88) (38),.

#### 2.5.6 Childbirth outcomes

Perinatal outcomes were categorized into maternal pregnancy and neonatal outcomes. Maternal outcomes were measured by gestational age and incidences of postpartum hemorrhage, pre-eclampsia, placenta previa, and premature rupture of membranes. Neonatal outcomes included birth weight, 1-min Apgar score, and incidences of prematurity, low birth weight, and neonatal asphyxia. Low birth weight was considered present if either twin had a birth weight below 2500g. Maternal outcomes were extracted from the hospital electronic records, while neonatal outcomes were assessed by board-certified pediatricians with extensive clinical experience and standardized training in neonatal assessment. All assessors were blinded to group allocation.

### 2.6 Statistical analysis

Data analysis was conducted using SPSS 26.0. The primary statistical method used was repeated measures analysis of variance (RM-ANOVA), which was employed to assess changes in FFMQ, PSS, STAI-S), and EPDS scores across three time points: pre-intervention (T1), post-intervention (T2), and 1-month post-delivery (T3). To ensure robust results, a *post hoc* intention-to-treat (ITT) analysis was conducted. We used the Last Observation Carried Forward (LOCF) method to impute missing data. Odds Ratios (OR) are used for dichotomous outcomes, while Difference in Means (DM) is used for continuous outcomes. A p-value of < 0.05 was considered statistically significant.

## 3 Results

### 3.1 General information in intervention group and control group

A total of 120 participants were included in the study. The average age of the participants was 30.80 years (SD = 3.82), with a mean gestational age of 24 weeks. No significant differences were observed in these demographic characteristics between the intervention and control groups. There are no significant differences in FFMQ, PSS, EPDS, and STAI-S scores between the intervention and control groups (**Table 1**).

**Table 1:** General information and outcomes in intervention group and control group.

Variable	IG (n=60)	CG (n=60)
Maternal age (year)	30.28 ± 3.28	31.32 ± 4.26
Pre-gravid BMI (kg/m^2^)	22.65 ± 3.48	23.40 ± 3.49
Education		
≤Senior high school	14 (23.33%)	15 (25.00%)
>Senior high school	46 (76.67%)	45 (75.00%)
Residence		
City	54 (90.00%)	56 (93.33%)
Town	4 (6.67%)	3 (5.00%)
Rural	2 (3.33%)	1 (1.67%)
Previous health status		
Good	37 (61.67%)	33 (55.00%)
Worse	23 (38.33%)	27 (45.00%)
Depression history		
Yes	0 (0.00%)	3 (5.00%)
No	60 (100.00%)	57 (95.00%)
Parity		
Primiparous	47 (78.33%)	52 (86.67%)
Multiparous	13 (21.67%)	8 (13.33%)
Planned pregnancy		
Yes	54 (90.00%)	52 (86.67%)
No	6 (10.00%)	8 (13.33%)
Mode of conception		
Spontaneous conceived	27 (45.00%)	17 (28.33%)
Assisted reproduction	33 (55.00%)	43 (71.67%)
Type of pregnancy		
Dichorionic diamniotic	45 (75.00%)	47 (78.33%)
Monochorionic diamniotic	15 (25.00%)	13 (21.67%)
Monochorionic monoamniotic	0 (0.00%)	0 (0.00%)
Primary outcomes		
EPDS	7.70 ± 3.18	7.77 ± 3.23
FFMQ	128.63 ± 12.03	124.05 ± 14.30
PSS	34.23 ± 7.94	33.88 ± 7.95
STAI-S	35.33 ± 8.72	35.92 ± 8.50

IG, intervention group; CG, control group; BMI, Body Mass Index; FFMQ, The Five Facet Mindfulness Questionnaire; PSS, Perceived Stress Scale; EPDS, Edinburgh Postpartum Depression Scale; STAI-S, State Anxiety Inventory; M ± SD or n (%).

### 3.2 Between-group comparison of EPDS, FFMQ, PSS, and STAI-S in the intervention group and control group

Of the 120 participants, 109 (90.83%) completed the intervention and were included in per-protocol analysis. Among the 60 participants in the intervention group, 59 (98.33%) attended at least 5 of the 6 weeks, and 55 (91.67%) reported engaging in home practice at least 3 times per week during the intervention period.

**Figure 2:**
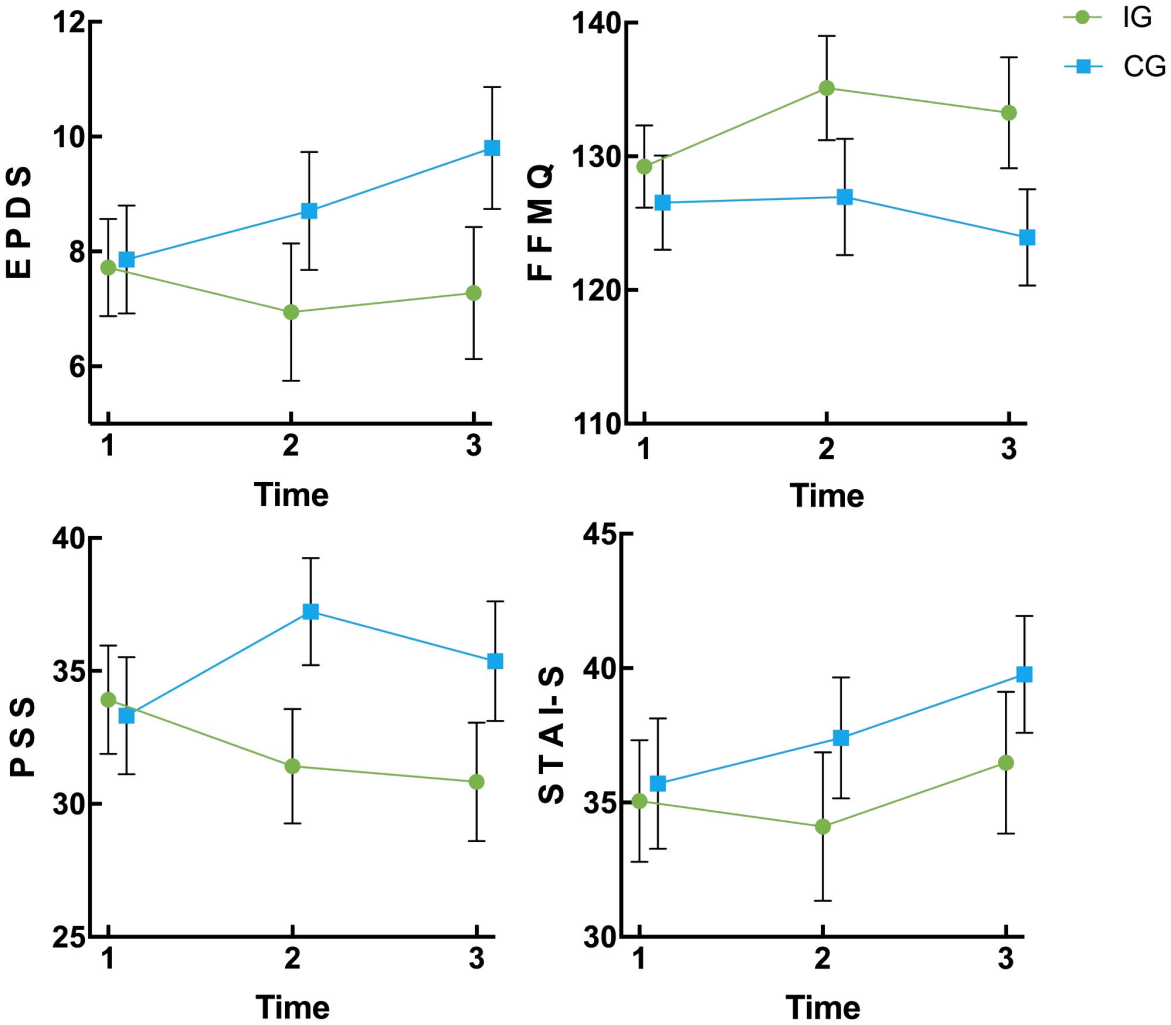
Changes of EPDS, FFMQ, PSS, and STAI-S in the intervention group and control group at three time points (95% CI) IG, intervention group; CG, control group; EPDS, Edinburgh Postpartum Depression Scale; FFMQ, The Five Facet Mindfulness Questionnaire; PSS, Perceived Stress Scale; STAI-S, State Anxiety Inventory; T_1_, pre-intervention; T_2_, post-intervention; T_3_, 1-month post-delivery; CI, confidence interval.

Repeated measures ANOVA was used to evaluate the differences in changes over time in FFMQ, PSS, EPDS, and STAI-S scores between the intervention group and the control group of women with twin pregnancies. Since the PSS and FFMQ scores did not meet the sphericity assumption of Mauchly’s test (P < 0.05), multivariate analysis of variance (MANOVA) was used for the analysis. According to the per-protocol analysis results, at T2 and T3, there were significant differences in EPDS, FFMQ, and PSS scores between the intervention group and the control group (P < 0.05), indicating that the intervention had a significantly positive effect in these areas; however, no significant difference was observed in STAI-S scores (P > 0.05). When comparing T2 with T1, there were significant differences in EPDS, FFMQ, PSS, and STAI-S scores between the two groups (P < 0.05). When comparing T3 with T1, there were significant differences in FFMQ, PSS, and EPDS scores (P < 0.05), while there was no significant difference in STAI-S scores (P > 0.05). Overall, this intervention has a significant effect on enhancing mindfulness, reducing perceived stress, and preventing the worsening of postpartum depression symptoms, but its impact on state anxiety is limited (**Table 2** and **Figure 2**).

**Table 2:** Between-group comparison of EPDS, FFMQ, PSS, and STAI-S in the intervention group and control group (per-protocol analysis).

Variable	Time	IG (n=58)	CG (n=51)	MD	95% CI	p-value
EPDS	T2	6.95 ± 4.55	8.71 ± 3.66	-1.76	[-3.33, -0.19]	0.030*
	T2-T1	-0.77	+0.85	-1.62	[-2.89, -0.35]	0.013*
	T3	7.28 ± 4.38	9.80 ± 3.77	-2.52	[-4.07, -0.97]	0.002*
	T3-T1	-0.44	+1.94	-2.38	[-3.65, -1.13]	<0.001*
FFMQ	T2	135.10 ± 14.91	126.96 ± 15.51	8.14	[2.41, 13.87]	0.010*
	T2-T1	+5.86	+0.43	5.43	[1.24, 9.62]	0.012*
	T3	133.26 ± 15.76	123.94 ± 12.86	9.32	[3.94, 14.70]	0.001*
	T3-T1	+4.02	-2.59	6.61	[2.43, 10.78]	0.002*
PSS	T2	31.41 ± 8.17	37.23 ± 7.17	-5.82	[-8.70, -2.94]	<0.001*
	T2-T1	-2.50	+3.92	-6.42	[-9.31, -3.54]	<0.001*
	T3	30.83 ± 8.50	35.37 ± 8.00	-4.54	[-7.64, -1.44]	0.010*
	T3-T1	-3.08	+2.06	-5.14	[-8.40, -1.89]	0.002*
STAI-S	T2	34.10 ± 10.52	37.41 ± 8.03	-3.31	[-6.90, 0.29]	0.071
	T2-T1	-0.95	1.71	-2.66	[-5.24, -0.07]	0.044*
	T3	36.48 ± 10.04	39.76 ± 9.15	-3.28	[-6.72, 0.16]	0.061
	T3-T1	1.43	4.06	-2.63	[-5.65, 0.40]	0.088

*: p-value < 0.05; IG, intervention group; CG, control group; FFMQ, The Five Facet Mindfulness Questionnaire; PSS, Perceived Stress Scale; EPDS, Edinburgh Postpartum Depression Scale; STAI-S, State Anxiety Inventory; T_1_, pre-intervention; T_2_, post-intervention; T_3_, 1-month post-delivery; MD, mean difference; CI, confidence interval; M ± SD

Through intention-to-treat analysis (ITT), it was found that there was a significant difference in the change scores of EPDS between the intervention group and the control group from T1 to T2 (P < 0.05), indicating that even considering participants who did not complete the entire study, the intervention still had a significant effect on reducing postpartum depression symptoms (**Table 3**).

**Table 3:** Between-group comparison of EPDS, FFMQ, PSS, and STAI-S in the intervention group and control group from ITT (ITT Analysis with LOCF Imputation).

Variable	Time	IG (n=60)	CG (n=60)	MD	95% CI	p-value
EPDS	T2	6.98 ± 4.49	8.57 ± 3.48	-1.59	[-3.04, -0.13]	0.033*
	T2-T1	-0.72	0.80	-1.52	[-2.67, -0.36]	0.011*
	T3	7.30 ± 4.32	9.50 ± 3.63	-2.20	[-3.64, -0.76]	0.003*
	T3-T1	-0.40	1.73	-2.13	[-3.29, -0.98]	<0.001*
FFMQ	T2	134.33 ± 15.25	125.12 ± 16.39	9.21	[3.49, 14.94]	0.002*
	T2-T1	5.70	1.07	4.63	[0.46, 8.81]	0.030*
	T3	132.55 ± 15.97	122.55 ± 14.01	10.00	[4.57, 15.43]	<0.001*
	T3-T1	3.92	-1.5	5.42	[1.22, 9.61]	0.012*
PSS	T2	31.72 ± 8.43	37.83 ± 7.06	-6.12	[-8.93, -3.31]	<0.001*
	T2-T1	-2.51	3.95	-6.47	[-9.19, -3.74]	<0.001*
	T3	31.15 ± 8.75	36.25 ± 7.93	-5.10	[-8.12, -2.08]	0.001*
	T3-T1	-3.08	2.37	-5.45	[-8.50, -2.40]	<0.001*
STAI-S	T2	34.45 ± 10.58	37.48 ± 7.85	-3.03	[-6.40, 0.34]	0.770
	T2-T1	-0.88	1.56	-2.44	[-4.80, -0.10]	0.040*
	T3	36.75 ± 10.05	39.48 ± 7.64	-2.73	[-5.96, 0.49]	0.100
	T3-T1	1.42	3.56	-2.14	[-4.91, 0.61]	0.130

ITT, intention-to-treat; LOCF, last observation carried forward; *, p-value < 0.05; IG, intervention group; CG, control group; EPDS, Edinburgh Postpartum Depression Scale; FFMQ, The Five Facet Mindfulness Questionnaire; PSS, Perceived Stress Scale; STAI-S, State Anxiety Inventory; T_1_, pre-intervention; T_2_, post-intervention; T_3_, 1-month post-delivery; CI, confidence interval; MD, mean difference; M ± SD.

### 3.3 Childbirth outcomes in intervention group and control group

In twin pregnancies, the first newborn refers to the baby delivered first, and the second newborn refers to the baby delivered second. The incidence of low-birth-weight infants was significantly lower in the intervention group compared to the control group (P<0.05). However, no significant differences were observed between the two groups for other outcomes (**Table 4**).

**Table 4:** Comparison of perinatal outcome in the intervention and control groups.

Outcomes	IG(n=58)	CG(n=51)	MD/OR	95% CI	p-value
Gestational week of labor	36.09 ± 1.94	36.12 ± 1.84	-0.29 ^a^	[-0.75, 0.69]	0.94
First-born twin					
Neonatal weight (g)	2397.40 ± 468.55	2411.96 ± 353.85	-14.56 ^a^	[-173.97, 144.84]	0.86
1min Apgar score	9.21 ± 1.27	9.63 ± 0.69	-0.42 ^a^	[-0.80, -0.04]	0.03
Second-born twin					
Neonatal weight (g)	2339.48 ± 434.67	2296.76 ± 356.71	43.41 ^a^	[-110.21, 197.03]	0.58
1min Apgar score	8.88 ± 1.24	9.21 ± 1.29	-0.24 ^a^	[-0.73, 0.25]	0.33
Prematurity	31 (53.45%)	28(54.90%)	0.94^b^	[0.44, 2.01]	0.88
Low birth weight	38 (65.52%)	44(86.27%)	0.30 ^b^	[0.12, 0.79]	0.01*
Postpartum hemorrhage	1 (1.72%)	0 (0)	/		1.00
Neonatal asphyxia	2 (3.44%)	1(1.96%)	1.79 ^b^	[0.16, 20.30]	1.00
Pre-eclampsia	1 (1.72%)	1(1.96%)	0.88 ^b^	[0.05, 14.39]	1.00
Placenta previa	2 (3.44%)	1(1.96%)	1.79 ^b^	[0.16, 20.30]	1.00
Prelabor rupture of membranes	12 (20.69%)	5(9.80%)	2.4 ^b^	[0.78, 7.36]	0.12

IG, intervention group; CG, control group; ^a^, MD mean difference; ^b^, OR odds ratio; CI, confidence interval; *, p-value < 0.05;M ± SD or n (%).

## 4 Discussion

Twin pregnancies are considered high-risk due to their association with a wide range of adverse birth outcomes and complications (39). Additionally, maternal depression is known to negatively affect perinatal outcomes (40). Our study found that group mindfulness therapy effectively alleviated depressive symptoms in women with twin pregnancies. Furthermore, the preventive benefits of the mindfulness intervention, initiated in early pregnancy, persisted from the third trimester to one month postpartum. Notably, the mindfulness intervention also appeared to reduce the incidence of low-birth-weight infants in twin pregnancies. To the best of our knowledge, this study is the first to design and implement a mindfulness intervention specifically tailored for twin pregnancies. It is also the first to explore the impact of a mindfulness intervention on pregnancy outcomes in this high-risk population.

After 6 weeks of group mindfulness intervention, a significant difference in depression scores was observed between the intervention and control groups, but no significant differences in the comparison from baseline to post-intervention. These findings suggest that the group mindfulness intervention helped prevent the worsening of depressive symptoms among women with twin pregnancies. Previous research has primarily focused on the impact of mindfulness interventions on singleton pregnancies. Research by Corbally & Wilkinson found that mindfulness interventions significantly enhance mindfulness and decrease depressive symptoms during pregnancy, especially in women without pre-existing mental health conditions (41). Similarly, a recent systematic review showed that mindfulness-based interventions (MBIs) significantly reduced depression in singleton pregnancies, with mindfulness-based cognitive therapy (MBCT) showing stronger efficacy in reducing depressive symptoms (23). Compared to these previous studies, our findings suggest that mindfulness intervention has a relatively insignificant impact on depressive symptoms in twin pregnancies. This difference highlights the unique challenges associated with twin pregnancies and emphasizes the need for tailored approaches to address their psychological and physical demands.

Several factors might explain the relatively smaller effect observed in our study compared to previous research. The primary reason is likely the higher prevalence and severity of psychological depression in twin pregnancies compared to singleton pregnancies. The unique stressors and complexities associated with twin pregnancies, such as increased physical demands and higher medical risks, can significantly elevate psychological distress. Studies indicate that approximately one-third of women with twin pregnancies experience depressive symptoms, almost twice the rate observed in singleton pregnancies (7, 42). Another contributing factor is the more significant physical changes that women carrying twins undergo. These challenges, such as fatigue and discomfort, can hinder their ability to concentrate and fully engage in mindfulness practices (43). Finally, the mindfulness group therapy (MGT) in this study was conducted online and with a relatively short duration. Although online interventions improve accessibility, they may lack the interpersonal engagement and immersive nature of in-person programs. Furthermore, shorter interventions may be less effective than longer, more intensive mindfulness programs. These considerations emphasize the need for more tailored and comprehensive mindfulness interventions to address the specific challenges faced by women with twin pregnancies.

The beneficial effects of mindfulness extended into the early postpartum period, consistent with findings from studies on singleton pregnancies (44). As pregnancy progresses, psychological challenges intensify (45), and depressive symptoms become more prevalent after childbirth (46, 47). Notably, depressive symptoms during pregnancy are linked to postpartum depressive trajectories, increasing the risk of postpartum depression and placing vulnerable individuals at greater risk (48). Mindfulness practices, such as breathing exercises, are accessible, practical, and easily integrated into daily life (49). Even after giving birth, women can independently use these positive thinking techniques to reduce stress and enhance their mindset. Ongoing engagement in mindfulness practices can significantly alleviate postnatal depressive symptoms in women with twin pregnancies. Furthermore, participation in MGT may foster social interaction and support among women carrying twins (50). These supportive relationships, established during pregnancy, extend into the postpartum period, facilitating the sharing of postnatal challenges and providing reciprocal comfort. Such social networks are vital for the prevention and mitigation of postpartum depressive symptoms.

The findings of this study revealed that the prevalence of low-birth-weight infants was significantly lower in the intervention group compared to the control group, aligning with previous research outcomes. For instance, a recent systematic review and meta-analysis by Laura et al. demonstrated that various stress-reducing interventions significantly decreased the rate of low birth weight (51). Similarly, Francesca et al. found that a combination of the Mediterranean diet and mindfulness intervention in pregnant women at high risk of SGA significantly reduces the percentage of newborns with birth weights below the 10th percentile (52). Narendran et al. also reported that yoga, a kind of mindfulness training, improved birth weights, with a significantly higher proportion of newborns exceeding 2500 grams in the intervention group (53).

However, our study did not observe significant differences between the intervention and control groups in other outcomes, such as gestational age at delivery, fetal birth weight, or adverse perinatal events like postpartum hemorrhage and neonatal asphyxia. Several factors could explain these findings. Firstly, all participants received regular antenatal check-ups and standardized care at the same hospital, which may have mitigated severe adverse outcomes. Secondly, the heterogeneity in intervention approaches—such as differences in the design and delivery of mindfulness interventions compared to other studies—could have influenced the outcomes. Finally, as noted by Santana et al., maternal complications in twin pregnancies are independent risk factors for adverse perinatal outcomes (54), which may have limited the impact of MGT on these outcomes. These findings highlight the need for further research, including longer offline mindfulness interventions, randomized controlled designs with paired samples, and studies that explore the influence of maternal complications.

This study has several limitations. Firstly, MGT follows a principle-based approach, which may lead to variations in its implementation across studies. Secondly, conducting the intervention online restricted participants’ access to face-to-face interaction with therapists, reducing opportunities for personalized guidance and tailored support. Thirdly, the single-blind randomized controlled design may have introduced biases, as the intervention group received more attention than the control group. This discrepancy could affect the overall quality of the methodology quality and introduce confounding factors.

## 5 Conclusion

A 6-week, 2-hours per week mindfulness group therapy (MGT) program appears to be effective in reducing perinatal stress and preventing the exacerbation of prenatal depression in women pregnant with twins. The beneficial effects of group mindfulness intervention during early pregnancy persist from the third trimester through to one month postpartum. Additionally, mindfulness intervention may reduce the incidence of low-birth-weight infants in twin pregnancies. Further studies are needed to validate these findings and explore the broader impact of mindfulness interventions on maternal and neonatal outcomes in twin pregnancies.

## Data Availability

The raw data supporting the conclusions of this article will be made available by the authors, without undue reservation.
