# Supplementary material for: Effect of mindfulness group therapy on maternal psychological distress and perinatal outcomes in twin pregnancy: a randomized controlled trial
Source: Front Psychiatry. 2025 May 20;16:1572633. doi: 10.3389/fpsyt.2025.1572633 (PMC12130052; doi:10.3389/fpsyt.2025.1572633)
Supplement: Supplementary file 1 [file Table1.docx]

Appendix 1 the curriculum of group mindfulness-based intervention for women pregnant with twins

| **Thesis** | **Group activities** | **Mian content of lecture** | **Formal**  **mindfulness practice** | **Informal**  **mindfulness practice** | **Homework** |
| --- | --- | --- | --- | --- | --- |
| Maternal Mindfulness Fundamentals | 1.Secret Angel  2. Snowball Self-Introduction | 1. What is Mindfulness？  2. Benefits of Mindfulness  3. Seven Principles of Mindfulness Practice  4. Objectives and Significance of this Course | 1. Mindful Seated Yoga Stretching  2. Sitting Practice: Mindfulness Meditation and Mindful Breathing Exercise | Brief Awareness of Surroundings and Body Sensations | 1. Reading the Article "Non-Judgment"  2. Daily Mindfulness Practice Handbook |
| Embracing Change, Mindful Conception | 1. Uncomfortable Sitting Experience  2. Relaxation Training | 1. Physical Changes and Discomforts in Expecting Mothers of Twins  2. Mindfulness and Physiological Discomforts During Pregnancy | 1. Mindful Lying Down Practice  2. Body Scan: Noticing Bodily Sensations | Mindful Pregnancy Discomfort Awareness Practice: Being Present with Discomfort | 1. Reading the Article "Acceptance"  2. Daily Mindfulness Practice Handbook |
| Focusing on the Present, Enjoying Mindfulness | Raisin Meditation | 1. The Power of Focus During Pregnancy  2. Letting Go in Mindfulness | 1. Mindful Standing Stretching  2. Standing Practice: Mindfulness Meditation | 1.Mindful eating  2.Mindful walking | 1. Reading the Article "Non-striving"  2. Integrating Mindfulness into Daily Life  3. Posting Mindfulness Reminder Posters |
| Mindfulness for Coping with Negative Emotions During Pregnancy | 1.Circle of Stress  2. 9-Dots Exercise | 1. Recognizing the Inertia of Thought  2. Breaking Free from Fixed Thought Patterns  3. Labeling Negative Emotions During Pregnancy | 1. Seated Yoga Stretching  2. Sitting Practice: Noticing Thoughts and Emotions | Momentary Pause Practice | 1. Reading the Article "Letting Go"  2. Joyful Event Recording Practice  3. Unpleasant Event Recording Practice |
| Mindful Interpersonal Communication During Pregnancy | Simulated Meditation Conversation | 1. Mindful Awareness in Interpersonal Communication  2. Your Baby, Your Mindfulness Teacher | 1. Mindfulness Meditation Practice  2. Sitting Practice: Noticing Sounds | Mindfully Listening and Speaking Without Judgment | 1. Reading the Articles "Trust" and "Patience"  2. Communication Difficulty Event Log |
| Mindfulness has no endpoint, Endings are also beginnings | 1. Revealing the Secret Angel  2. Gift Delivery | 1. Exploring and Clarifying the Concerns  2. Sharing Insights from Mindfulness Practice During Pregnancy  3. How to Cultivate the Habit of Mindfulness Practice | 1. Mindful Standing Yoga Stretching  2. Mindful Kegel Exercises  3. Embracing Challenges and Committing to Long-term Plans | Mindful Self-Compassion: Sending Compassion to Yourself and Your Baby | 1. Reading the Article "Beginner's Mind"  2. Continuing Mindfulness Practice |
